# Differential expression pattern of the proteome in response to cadmium stress based on proteomics analysis of wheat roots

**DOI:** 10.1186/s12864-020-6716-8

**Published:** 2020-05-07

**Authors:** Mingyang Jian, Dazhong Zhang, Xiaoying Wang, Shuwei Wei, Yue Zhao, Qin Ding, Yucui Han, Lingjian Ma

**Affiliations:** 1 College of Agronomy, Northwest A&F University, Yangling, 712100 China; 2 College of Horticulture, Northwest A&F University, Yangling, 712100 China

**Keywords:** Cd stress, Wheat roots, Chemical forms, Proteomics analysis

## Abstract

**Background:**

Heavy metal cadmium (Cd) is a common environmental pollutant in soils, which has an negative impacts on crop growth and development. At present, cadmium has become a major soil and water heavy metal pollutant, which not only causes permanent and irreversible health problems for humans, but also causes a significant reduction in crop yields.

**Results:**

This study examined the chemical forms of Cd in the roots of two wheat varieties (M1019 and Xinong20) by continuous extraction and analyzed differences in distribution characteristics of Cd in the root cell wall, cytoplasm, and organelles by elemental content determination and subcellular separation. Furthermore, we conducted proteomics analysis of the roots of the two varieties under Cd pollution using mass spectrometry quantitative proteomics techniques. A total of 11,651 proteins were identified, of which 10,532 proteins contained quantitative information. In addition, the differentially expressed proteins in the two varieties were related to DNA replication and repair, protein metabolism, and the glutathione metabolism pathway.

**Conclusion:**

The results of this study improve our understanding of the mechanism of plant responses to Cd stress.

## Background

The harmful effects of nonliving factors on organisms in a specific environment are called abiotic stress [1]. In the natural environment, abiotic stresses including heat, cold, and heavy metals are not only harmful to the environment, but also detrimental to plants and agriculture [2, 3]. Recent studies have shown that environmental pollution, such as heavy metal pollution, poses a serious threat to living organisms. Plants can cause dysfunction of plant proteins under heavy metal stress [4]. With the intensification of human activities such as mining and industrial activities and the excessive using of phosphate fertilizer, cadmium is released into the natural environment through geological and human activities. Even at low concentrations, cadmium is one of the most toxic elements, which is due to high fluidity and bioavailability. Therefore, cadmium can more easily be absorbed in the underground part of plants and accumulated in the aerial part [5, 6].

Wheat is one of the most important and the foremost food crops in China. In 2011–2012, wheat production was significantly higher than corn and rice (USDA 2011). Globally, the main food source for human protein intake is wheat, which has higher protein content than that of corn and rice [7, 8]. Cd is a toxic heavy metal that is absorbed by the roots, subsequently inhibiting plant growth and development. Roots are the first organs that are exposed to Cd ions. Studies have shown that the first defense strategy is to exclude the metal entering the root tissues. Roots rapidly respond to the presence of Cd by forming a mechanical barrier [9, 10]. Cadmium, absorbed by roots and accumulated in plants, may lead to physiological, biochemical and structural changes in plants such as free radicals, which directly cause cell peroxidation. It would influence photosynthetic and stimulates leaf apoptosis and necrosis [11–13]. In order to respond cell peroxidation, cadmium can be detoxified by phytochelatin (a glutathione-derived peptide) or use antioxidant enzymes to degrade excess reactive oxygen species (ROS) [14, 15]. In conclusion, plants adapt to adverse conditions through a series of physiological, cellular, and molecular processes, culminating in stress tolerance [16]. Extensive studies have been conducted on the physiological aspects of plant stress responses, whereas proteomics investigations, particularly involving wheat roots, are limited.

In order to better explain the resistance mechanism of wheat stress on cadmium, proteomics as a useful analysis tools have been used to analyze protein changes due to changes in environmental factors. Proteins are the end products of genes and play an important role in plant cell metabolism and biological processes. With the deepening of proteomics research, they have made an important supplement to gene expression research.

## Results

### Distribution of cadmium and DEPs subcellular structure localization

**Fig. 1:**
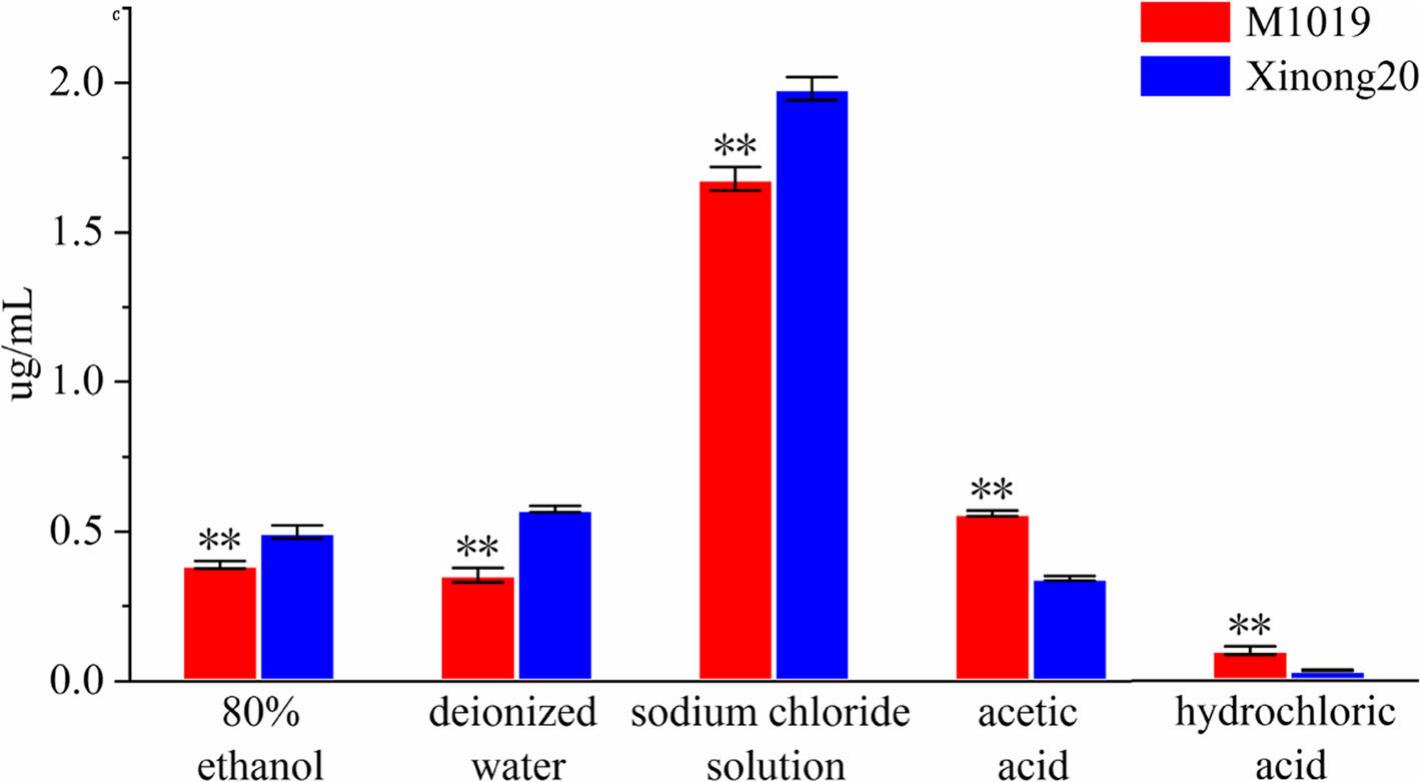
Cadmium chemical analysis After t-test, the significant differences at the level of 0.01

The combined form of M1019 and Xinong20 Cd is mainly based on the extracted state of sodium chloride (Fig. 1). The proportion of Cd in different chemical forms of M1019 variety was sodium chloride extraction state > acetic acid > extraction state > deionized water extraction state > ethanol extraction state> hydrochloric acid extraction state. The proportion of Cd in different chemical forms of Xinong20 variety is sodium chloride extraction state > deionized water extraction state > ethanol extraction state > acetic acid extraction state > hydrochloric acid extraction state.

**Fig. 2:**
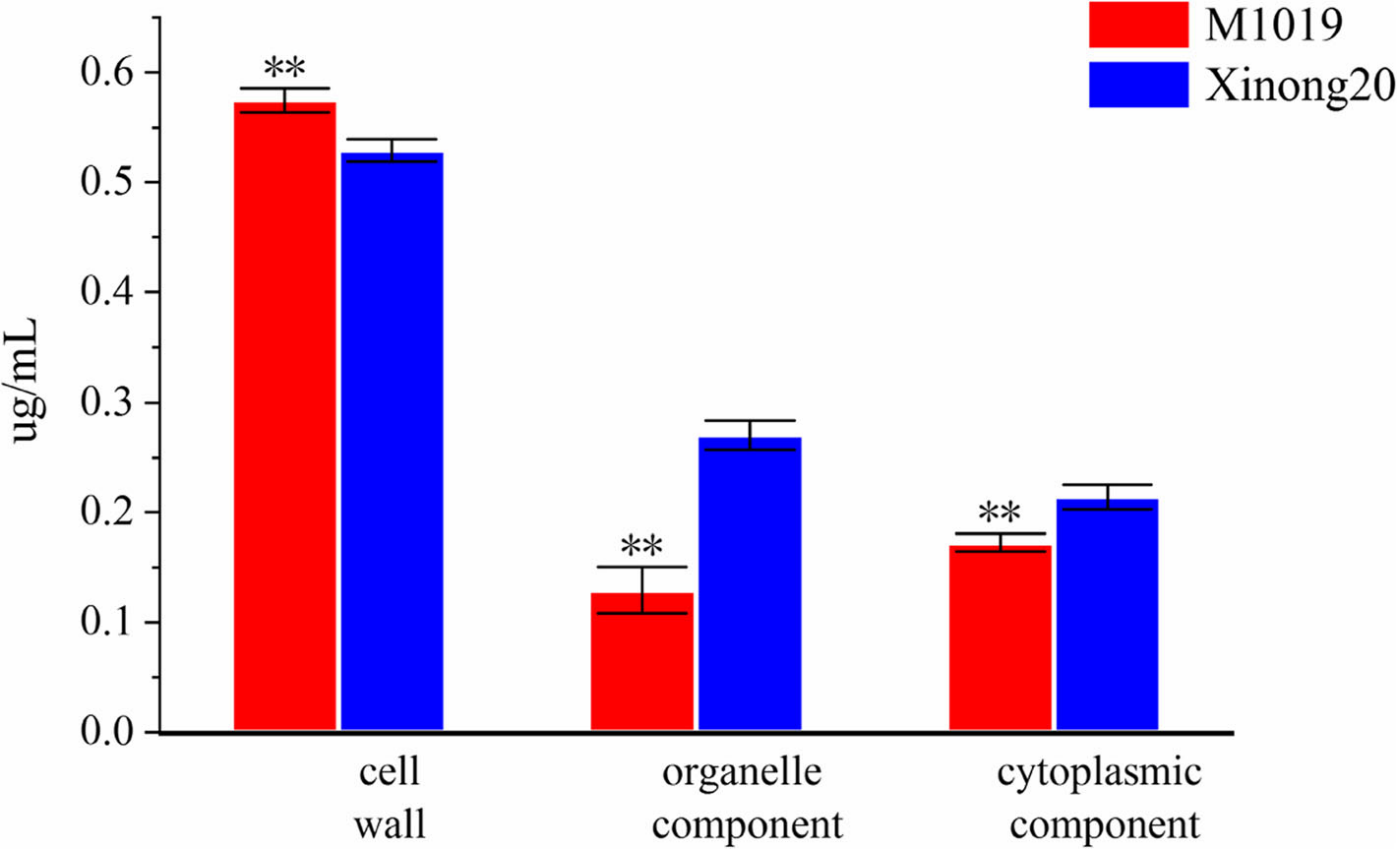
Distribution of cadmium in the cell wall, organelle components, and cytoplasmic After t-test, the significant difference at the level of 0.01

**Fig. 3:**
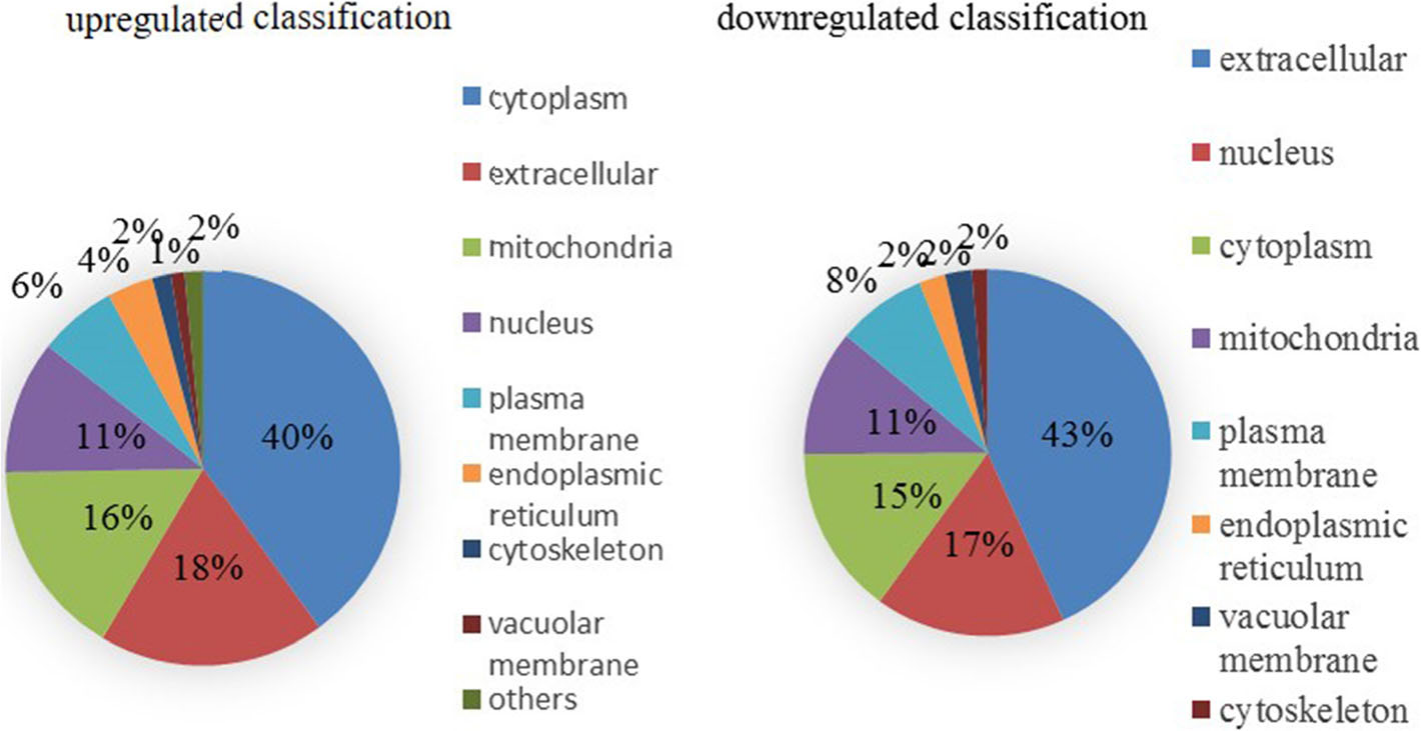
DAPs subcellular structure localization of M1019

**Fig. 4:**
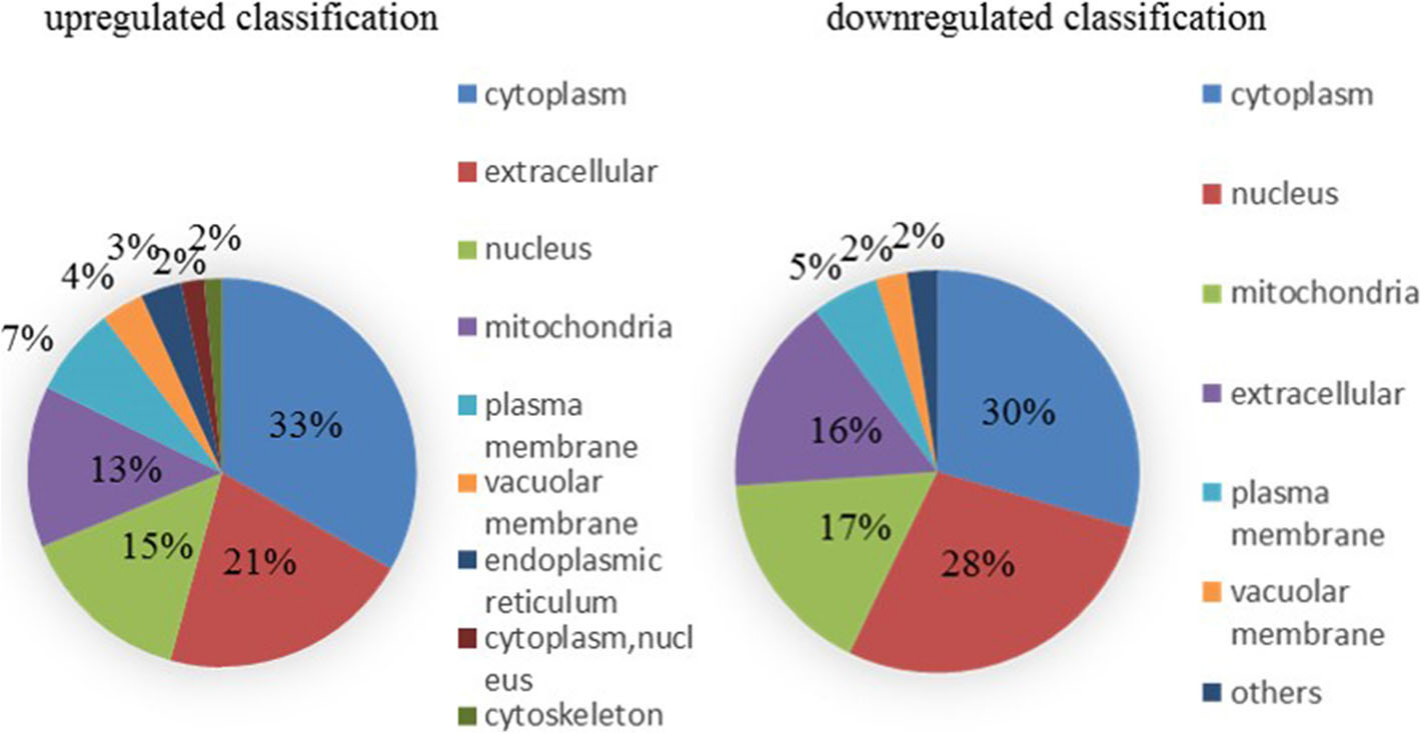
DAP subcellular structure localization of Xinong20

From the content of Cd of subcellular component (Fig. 2), Cd in root tissues of M1019 and Xinong20 was mainly distributed in the cell wall, however the Cd content of M1019 was the lowest in organelles, and the Cd content of Xinong20 was lowest in cytoplasmic. The original statement has been changed: The content of cadmium in the cell wall of M1019 was higher than that of xinong20, and the content of cadmium in the cell fluid and cytoplasm was lower than that of xinong20. In addition, we used wolfpsort, a subcellular localization prediction software, to predict the subcellular localization. The results showed that the upregulation protein that located in cytoplasm of M1019 is a little more than Xinong20. However, the downregulated M1019 located in cytoplasm was approximately half as much as Xinong20. (Figs. 3 and 4). The cytoplasm is the main site for biochemical reactions; therefore, Cd stresses may imparted greater effects on Xinong20. To further analyze the differentially expressed proteins, we conducted systematic bioinformatics analysis.

### Analysis of mass spectrometry data

**Fig. 5:**
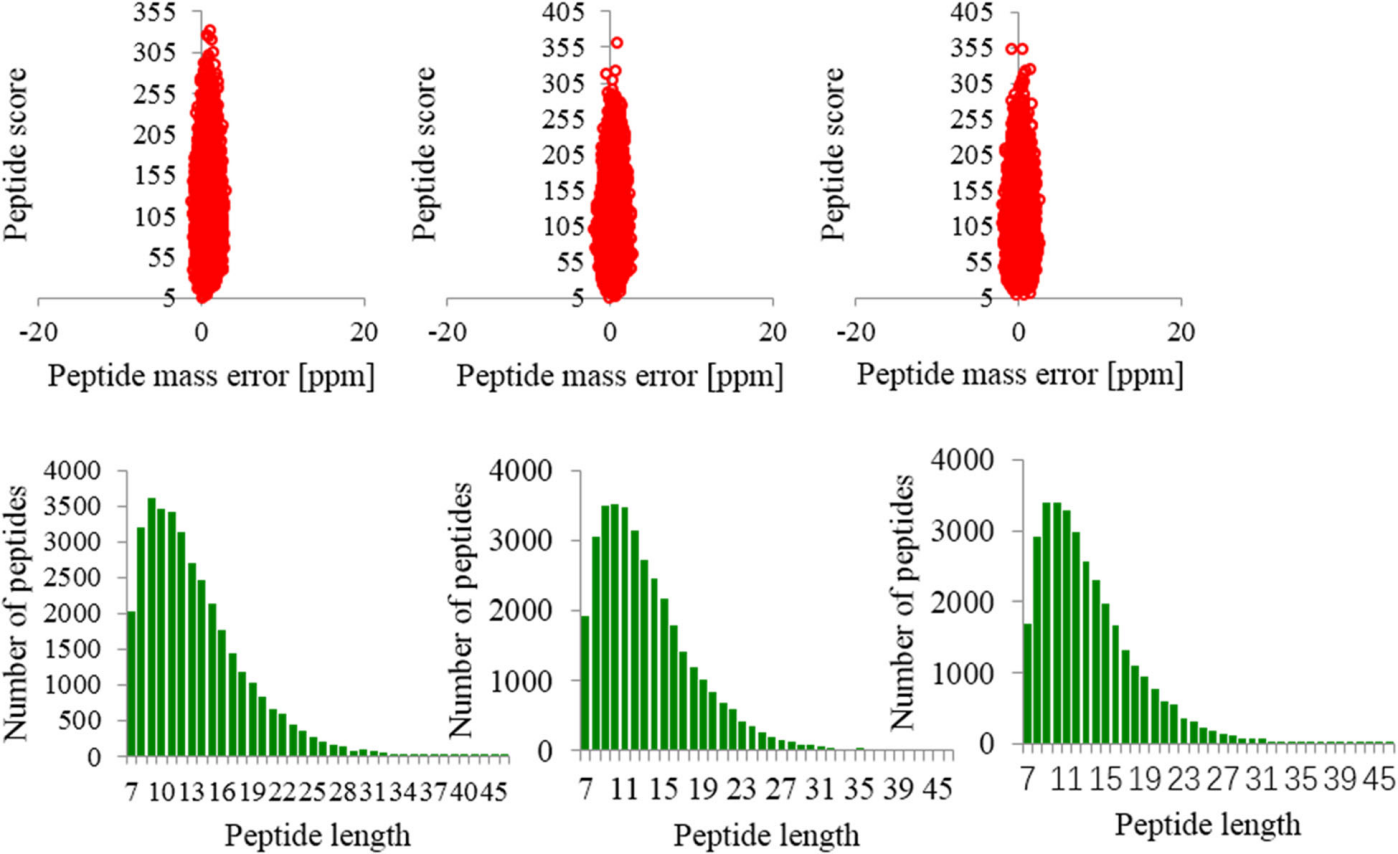
the mas error and peptide lengths of the sample. Three biological replications were performed for proteomics analysis of the samples, and the left, center and right respectively represents the result of the first, second and third replications

**Fig. 6:**
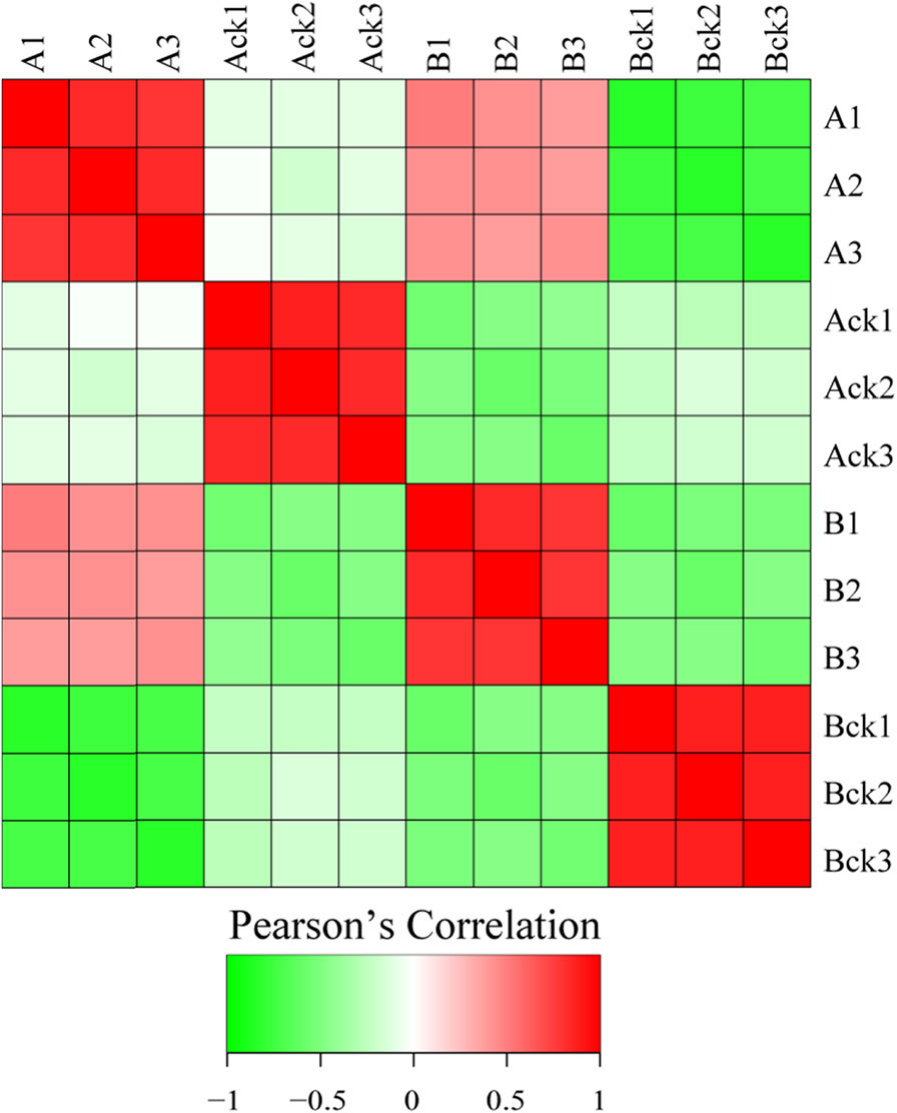
Heat map draw for calculating our Pearson’s Correlation Coefficient using all sample pairs. The A represents wheat M1019, and A1, A2, and A3 represent the three biological replications of cadmium treatment. Ack_1_, Ack_2_ and Ack_3_ represent three biological replications of the control (nutrient solution only without cadmium). The B represents wheat xinong20 and B1, B2 and B3 represent three biological replications of cadmium treatment. Bck_1_, Bck_2_ and Bck_3_ represent three biological replications of the control group (nutrient solution only without cadmium)

We conducted quality control testing of the obtained mass spectrometry data. First, mass errors in all identified peptides were assessed. The results showed that the quality error was centered at 0 and concentrated to a range below 10 PPM, indicating that the quality error met the requirements. Second, most of the peptide lengths were distributed between 7 and 16 amino acid residues (Fig. 5). The results showed that the sample conformed to the rule of and the sample preparation reached criterion, so the sample can be used for next step of biological information analysis. Pearson’s correlation coefficient was used to determine whether the biological repetition rate was statistically consistent. In this experiment, all sample pairs were used to calculate our Pearson’s correlation coefficient. The closer Pearson’s coefficient is a measure of the linear correlation between sets of data. When the coefficient closer to − 1 is a negative correlation, the closer to 1 is a positive correlation, the closer to 0 is relevant. The results (Fig. 6) showed that the three biological repeats were statistically consistent, indicating that the results were reliable.

A total of 11,651 proteins were identified, of which 10,532 contained quantitative information. Taking 1.5 times as the change threshold and t-test *p*-value< 0.05 as the standard, we determined that the expression of 268 proteins in M1019/M1019ck was upregulated and 217 proteins were downregulated. The expression of 684 proteins in Xinong20/Xinong20_ck_ was upregulated, and that of 680 proteins was downregulated.

### Gene ontology enrichment analyses of DEPs

**Fig. 7:**
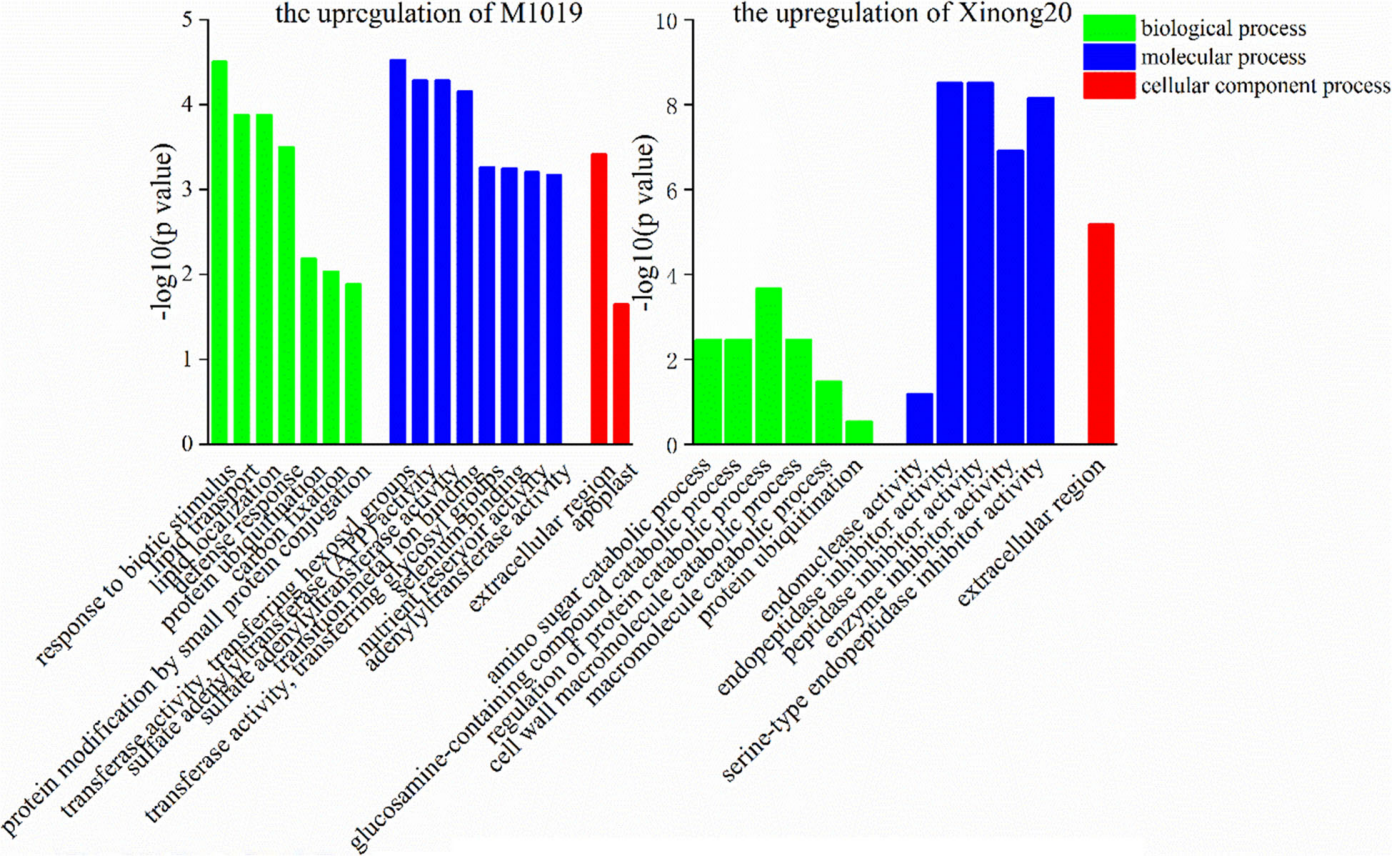
Gene Ontology (GO) analysis of the upregulation of M1019 and Xinong20

**Fig. 8:**
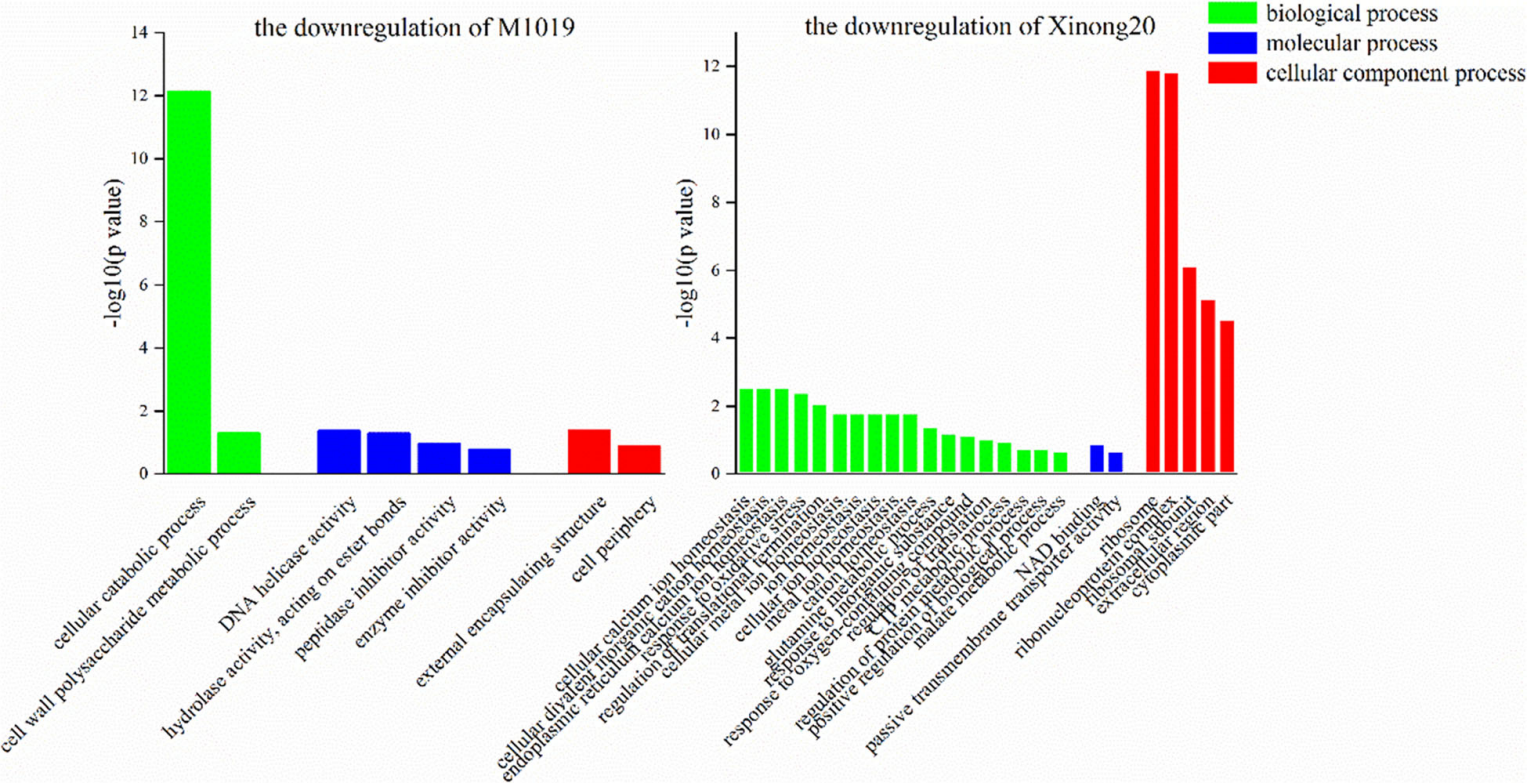
Gene Ontology (GO) analysis of downregulation of M1019 and Xinong20

We conducted GO annotation analysis on the identified differential proteins and explained the biological effects of proteins from different angles. Therefore, we conducted statistical analysis of the distribution of proteins quantified by M1019 and Xinong20 in the GO secondary annotations. The GO annotation is divided into three primary categories: the biological process, the cellular component, and the molecular function (Figs. 7 and 8). Among the biological process functions, the upregulated expression, the DEPs of M1019 were related to responses to stress and carbon fixation. The DEPs of Xinong20 were related to decomposition and amino sugar catabolic process, the cell wall macromolecule catabolic process, polysaccharide catabolic process, regulation of protein catabolic process, and protein ubiquitination. For downregulated expression, the DEPs of M1019 were related to cellular catabolic process. The differentially expressed proteins of Xinong20 were mainly related to the balance of the ion, cell metabolism, and macromolecular biosynthesis. Destruction of ion balance may be a factor that Xinong20 is sensitive to Cd stress.

Among the molecular process functions, the upregulated expression, the DEPs of M1019 were related to transferase activity, transferring glycosyl groups, and metal ion binding. The differentially expressed proteins of Xinong20 were mainly related to peptidase inhibitor activity, including those of endopeptidases. Inversely, the helicase activity of DNA and hydrolase activity, which act on ester bonds, enzyme inhibitor activity, and peptidase inhibitor activity were downregulated. Therefore, the difference in inhibiting enzyme activity will affect peptide synthesis in Xinong20 to some extent, which may indirectly affect protein metabolism process. Among the cellular component process functions, the upregulated expression, the DEPs of M1019 were related to intrinsic and integral components of the membrane. The DEPs of Xinong20 were related to the extracellular region. For the downregulated expression, the DEPs of M1019 were related to external encapsulating structure. The DEPs of Xinong20 were related to ribosomes and ribonucleoprotein complexes (Figs. 7 and 8).

### Genomes (KEGG) enrichment analysis of DEPs

**Fig. 9:**
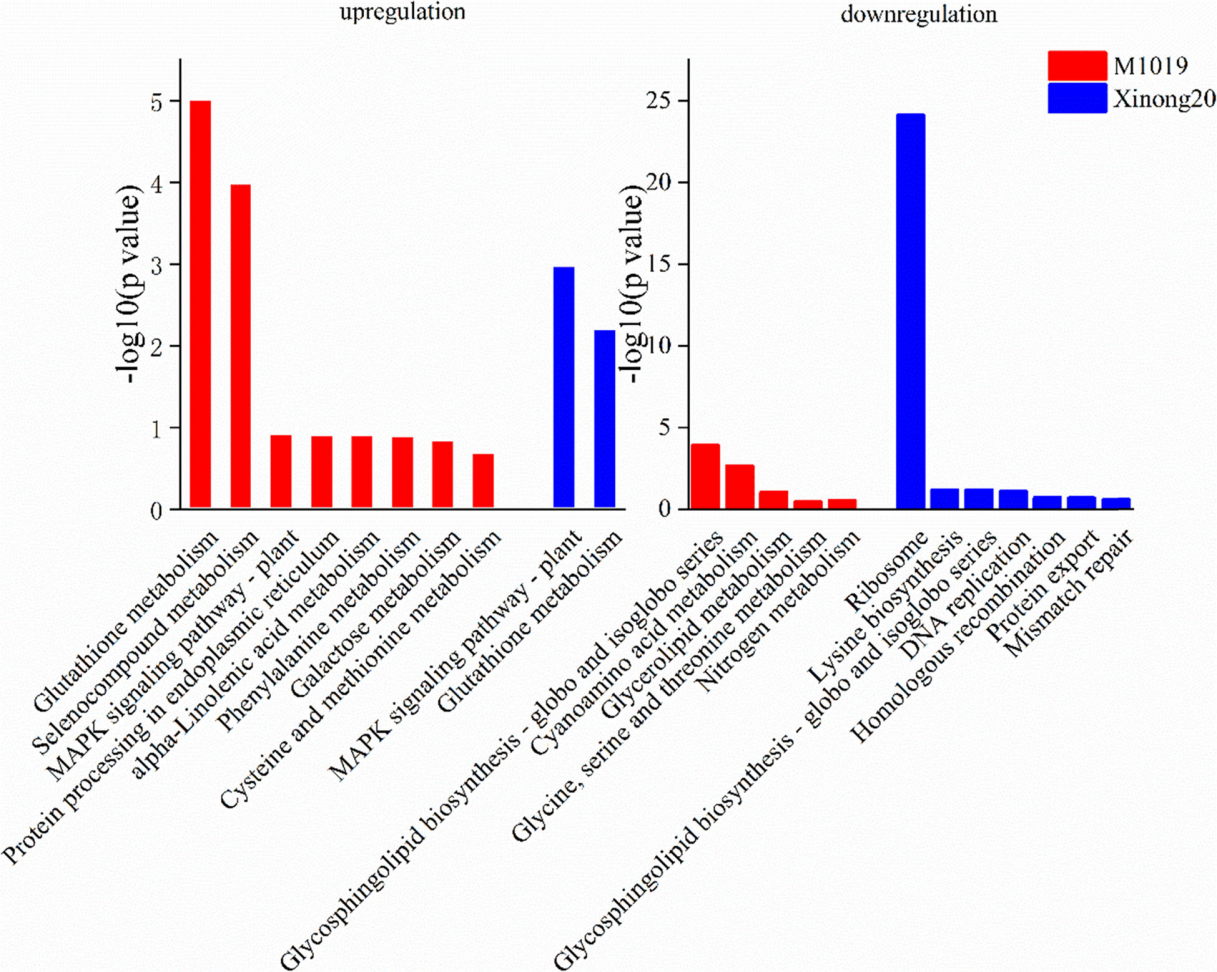
Main DAPs enriched in the pathways

We also made comparisons with the KEGG database to predict possibly important pathways. For upregulated expression, both M1019 and Xinong20 enriched the MAPK signaling pathway-plant and glutathione metabolism. However, the results showed that most of the DEPs in the MAPK signaling pathway of Xinong20 were related to pathogenic proteins. A significantly greater enrichment of the glutathione metabolism pathway of M1019 than that in Xinong20 was observed (Fig. 9). For the upregulated expression, the results indicated that the DEPs of M1019 also enriched in protein processing in endoplasmic reticulum, alpha-linolenic acid metabolism, phenylalanine metabolism, galactose metabolism, and cysteine and methionine metabolism (Table 1).

**Table 1:** The upregulation proteins in M1019 and Xinong20

Wheat cultivars	KEGG pathway	Protein description
M1019	Cysteine and methionine metabolism	Homocysteine S-methyltransferase 3
	Cysteine and methionine metabolism	1-aminocyclopropane-1-carboxylate oxidase
	Glutathione metabolism	Glutathione S-transferase 4
	Glutathione metabolism	Probable glutathione S-transferase GSTU6
	Glutathione metabolism	Protein IN2–1 homolog B
	Glutathione metabolism	Probable glutathione S-transferase GSTU6
	Glutathione metabolism	Probable glutathione S-transferase GSTU6
	Glutathione metabolism	Glutathione S-transferase U18
	Glutathione metabolism	Protein IN2–1 homolog B
	Glutathione metabolism	Protein IN2–1 homolog B
	Glutathione metabolism	Probable glutathione S-transferase GSTU6
	Glutathione metabolism	Probable glutathione S-transferase GSTU6
	Glutathione metabolism	Probable glutathione S-transferase GSTU6
	Glutathione metabolism	Galactinol--sucrose galactosyltransferase
	Glutathione metabolism	Hexokinase-7
	Protein processing in endoplasmic reticulum	Heat shock cognate 70 kDa protein
	Protein processing in endoplasmic reticulum	UDP-glucose:glycoprotein glucosyltransferase
	Protein processing in endoplasmic reticulum	17.8 kDa class II heat shock protein
	Protein processing in endoplasmic reticulum	Cell division cycle protein 48 homolog
Xinong20	Glutathione metabolism	Ornithine decarboxylase
	Glutathione metabolism	Glutamate--cysteine ligase A, chloroplastic
	Glutathione metabolism	Uncharacterized protein
	Glutathione metabolism	Probable glutathione S-transferase GSTU6
	Glutathione metabolism	Probable L-ascorbate peroxidase 3
	Glutathione metabolism	5-oxoprolinase
	MAPK signaling pathway - plant	Nucleoside diphosphate kinase 1
	MAPK signaling pathway - plant	Pathogenesis-related protein PRB1–3
	MAPK signaling pathway - plant	Pathogenesis-related protein PRMS
	MAPK signaling pathway - plant	Pathogenesis-related protein PRB1–2

In terms of downregulated expression, more pathways in Xinong20 were enriched than that observed in M1019. The DEPs related to glycosphingolipid biosynthesis-globo and isoglobo series were highly enriched in M1019 and Xinong20. Moreover, the DEPs related to nitrogen metabolism were enriched in M1019 such as the glutamine synthetase cytosolic isozyme. The DEPs were related to DNA replication, homologous recombination, mismatch repair, and ribosomes. Lysine biosynthesis, nitrogen metabolism, and protein export were also enriched in Xinong20. In terms of protein export pathways, the DEPs in Xinong20 were associated with transport proteins such as the mitochondrial inner membrane protein and signal recognition particle 54 kDa. For the ribosome pathway, numerous DEPs were enriched in Xinong20. In addition, the DNA polymerase delta small subunit and replication protein 32 kDa subunit showed downregulated in the DNA replication, mismatch repair, and homologous recombination pathway (Table 2).

**Table 2:** The downregulation proteins in M1019 and Xinong20

Wheat cultivars	KEGG pathway	Protein description
M1019	Glycerolipid metabolism	Alpha-galactosidase
	Glycosphingolipid biosynthesis - globo and isoglobo series	Alpha-galactosidase
	Glycosphingolipid biosynthesis - globo and isoglobo series	Beta-hexosaminidase 3
	Glycosphingolipid biosynthesis - globo and isoglobo series	Alpha-galactosidase
	Starch and sucrose metabolism	Beta-xylosidase/alpha-L-arabinofuranosidase 2
	Starch and sucrose metabolism	Beta-xylosidase/alpha-L-arabinofuranosidase 2
	Starch and sucrose metabolism	Beta-xylosidase/alpha-L-arabinofuranosidase 2
	Starch and sucrose metabolism	Beta-xylosidase/alpha-L-arabinofuranosidase 2
	Starch and sucrose metabolism	Endoglucanase 10
	Starch and sucrose metabolism	Beta-fructofuranosidase, insoluble isoenzyme 7
	Cyanoamino acid metabolism	Beta-xylosidase/alpha-L-arabinofuranosidase 2
	Cyanoamino acid metabolism	Beta-xylosidase/alpha-L-arabinofuranosidase 2
	Cyanoamino acid metabolism	Beta-xylosidase/alpha-L-arabinofuranosidase 2
	Cyanoamino acid metabolism	Beta-xylosidase/alpha-L-arabinofuranosidase 2
	Glycine, serine and threonine metabolism	Threonine dehydratase biosynthetic, chloroplastic
	Glycine, serine and threonine metabolism	Threonine dehydratase biosynthetic, chloroplastic
	Nitrogen metabolism	Glutamine synthetase cytosolic isozyme 1–3
	Nitrogen metabolism	Glutamine synthetase cytosolic isozyme 1–3
Xinong20	Protein export	Mitochondrial inner membrane protein OXA1
	Protein export	Signal recognition particle 54 kDa protein 1
	Protein export	Protein transport protein Sec61 subunit beta
	Protein export	Signal recognition particle 54 kDa protein 3
	DNA replication	DNA polymerase delta small subunit
	DNA replication	DNA replication licensing factor MCM4
	DNA replication	DNA replication licensing factor MCM5
	DNA replication	DNA polymerase delta small subunit
	Mismatch repair	Replication protein A 32 kDa subunit A
	Homologous recombination	DNA polymerase delta small subunit
	Homologous recombination	Replication protein A 32 kDa subunit A
	Homologous recombination	60S ribosomal protein L27a-3
	Ribosome	40S ribosomal protein S11
	Glycosphingolipid biosynthesis - globo and isoglobo series	Alpha-galactosidase
	Glycosphingolipid biosynthesis - globo and isoglobo series	Beta-hexosaminidase 3
	Lysine biosynthesis	Diaminopimelate epimerase, chloroplastic
	Spliceosome	Glycine-rich RNA-binding protein RZ1C
	Spliceosome	DEAD-box ATP-dependent RNA helicase 14
	Spliceosome	mediator of RNA polymerase II transcription subunit 37e
	Spliceosome	Heat shock cognate 70 kDa protein
	Spliceosome	Splicing factor U2af small subunit B

## Discussion

### Cd content distribution and chemical form distribution

Most of the heavy metal absorption takes place through root cells, which distribute toxic ions to specific organs, tissues, or organelles. Studies have shown that metal ions in Ni-rich plant cells, Zn hyperaccumulators and copper hyperaccumulators are mainly distributed on the cell wall, and the content in organelles is relatively small [17, 18]. In this study, the cadmium distribution of M1019 in root cells was the same as that in Ni-rich plant cells, Zn hyperaccumulators and copper hyperaccumulators, while the content of cadmium in cytoplasmic component of Xinong20 was higher than that in organelles. Therefore, M1019 can better accumulate cadmium in cell wall, which may be one of the factors of resistance to cadmium in M1019. In addition, the Cd content and percentage of cell organelles of M1019 were lower than Xinong20, and the Cd content and percentage of cell wall were higher than Xinong20. Therefore, M1019 can more effectively accumulate Cd in the cell wall and prevent excess Cd from entering cellular organelles. According to the GO analysis, among the cellular component process, for the downregulated expression, the DEPs were detected in cytoplasmic part. So Xinong20 may be more effected than M1019.

Studies have shown that absorption operations play an important role in maintaining trace amounts of cellular metal ions. Heavy metals can combine with polar compounds to form water-soluble, alcohol-soluble, and acid-soluble low-molecular weight metal chelates to affect heavy metals in plants [19]. Some studies indicate that the ethanol extraction state and water-soluble Cd have the strongest migration activity, and the acid-soluble (acetic acid and hydrochloric acid extraction) Cd had the weakest migration activity, so the Cd toxicity effect in the alcohol-soluble and water-soluble form is more significant than other chemical forms on Cd migration activity [20]. In this study, the content of Cd in the ethanol-extracted and water-extracted state of M1019 variety was lower, and the content of acid-soluble Cd with weaker migration activity was higher than Xinong20. Acidic extraction with weaker migration activity may also be more conducive to cadmium deposition on the cell wall, thereby slowing down Cd stress. However, the Cd with stronger migration activity is more likely to move upwards in Xinong20, thus causing toxicity to the plants. On the other hand, this may contribute to the accumulation of Cd in Xinong20 plants. We preliminarily concluded that the migration of Cd in M1019 is weaker than that in Xinong20, which may be a key factor for more cell wall deposition in M1019.

### Effect of cd stress on protein synthesis

Protein synthesis is substantially important for normal wheat development, new cell protein formation, protein degradation, and the output balance process. Proteins are synthesized via transcription, translation, post-translational processing, and modification, which finally lead to the synthesis of the mature protein, which is strictly regulated in multiple steps [21]. Nowadays, classical two-dimensional (2D) gel-based proteomics has been difficult to meet the analysis of large dynamic range due to several technical limitations including gel separation and identification capabilities [22], so we choose proteomics techniques to analysis.

Studies have found that Cd contamination can cause DNA damage to organisms such as DNA fragmentation and DNA cross-linking, which interferes with the normal expression of genes. In addition, Cd^2+^ can randomly bind to DNA, resulting in single-strand DNA breaks [23]. Most DNA damage due to metal toxicity involves DNA fragmentation. The MMR system is a major DNA repair pathway in all eukaryotes. Most errors that arise during DNA replication can be corrected by DNA polymerase proofreading or by post-replication MMR [24–26]. DNA recombinant repair protein can repair the damage caused by heavy metal pollution to the DNA molecules in the cells and restores its DNA structure and function. We found that DNA replication, homologous recombination, and mismatch repair pathway in Xinong20 were downregulated. DNA polymerase delta small subunit and replication protein A 32-kDa subunit showed downregulated in the three pathways. DNA polymerase and replication protein are essential to DNA replication. However, no decrease in these proteins was detected in M1019. Therefore, Cd stress will affect the normal replication of DNA and mismatch repair of Xinong20. DNA is a template for transcription. Thus, DNA replication and repair are disrupted, which is highly likely to cause errors in transcription.

After gene transcription, mRNA precursors contain protein-coding exons and noncoding introns and require splicing by spliceosomes before a mature mRNA is formed. We found that components of the spliceosome pathway were downregulated in Xinong20 such as splicing factor U2af small subunit B, serine /arginine-rich splicing factor RS31, and serine/arginine-rich SC35-like splicing factor SCL33. However, no downregulation was detected in M1019. The hn-RNA is the initial product of DNA transcription, which requires further processing and modification of the spliceosome to form mRNAs with coding function. Experimental results indicate that protein expression is further affected by processing of transcriptional primary products that affect mRNA of Xinong20.

Mitochondrial heat shock proteins play a role in the degradation of unstable and misfolded proteins and in the folding and refolding of proteins [27]. When cells are stressed by metal ions, heat shock proteins can treat harmful metal ions by helping other proteins fold. HSP70 on tomato cell membranes act as a molecular-chaperones that protect cells from damage [28]. We found that protein processing in the endoplasmic reticulum pathway was upregulated in M1019 such as heat shock cognate 70 kDa protein, 17.8 kDa class II heat shock protein, and cell division cycle protein 48 homolog. In contrast, the heat shock cognate 70 kDa protein and 17.8 kDa class II heat shock protein was downregulated in Xinong20. Heat shock proteins, which are involved in the translation process and in the post-processing process, were also downregulated in Xinong20. However, this was upregulated in M1019. By affecting the processing of Xinong20 mRNA post-transcriptional proteins, the synthesis of proteins is affected, ultimately affecting the expression of protein function. M1019 can effectively fold proteins to cope with Cd stress.

Approximately 30% of the eukaryotic proteome is transmitted to the downstream compartments by endoplasmic reticulum (ER) folding [29]. Signal recognition particles (SRPs) are important for targeting secreted proteins in a GTP dependent process, which transmit secreted proteins to the plasma membrane or endoplasmic reticulum [30]. This is critical to the vitality of all organisms [31, 32]. Studies have characterized canine SRP Srp72, Srp68, Srp54, Srpl9, Srpl4, and Srp9 based on their apparent molecular weights. In addition, the Sec61 complex is also the central component of the protein translocation apparatus of the ER membrane [33, 34].

In summary, proteins are products of gene expression. The rate of protein synthesis and transport was lower or abolished in Xinong20. We speculate that this may also be a key factor that indirectly affects the synthesis of macromolecular substances. This ultimately leads to the sensitivity of Xinong20 to Cd toxicity.

### Effect of cd stress on glutathione metabolism

GST plays an important role in plant stress tolerance, which can be induced under a variety of biotic or abiotic stress conditions, catalyzes the binding of glutathione to harmful substances, and transports the polymer to the vacuole through the action of ATP-binding transporter for detoxification [35]. Therefore, the binding and transport of glutathione to toxic substances usually require the participation of glutathione S-transferase (GST). On the other hand, glutamine synthetase (GS) is a key enzyme for plant-catalyzed conversion of glutamate (Glu) to glutamine (Gln). Decreased GS activity can promote the accumulation of Glu and contribute to the biosynthesis of the Cd-detoxifying substance, glutathione. In addition, ABC transporters in plants play a role in detoxification defense by transporting secondary metabolites. Numerous studies have revealed that ABC transporters are also required for normal plant development [36]. In this study, in the upregulation expression, the GST of M1019 and Xinong20 both showed an increase, but the M1019 showed a more increase. In the downregulation expression, the GS of M1019 showed a downregulation expression, but GS of Xinong20 did not show the downregulation expression. So, this will be beneficial for accumulating of glutathione. In addition, ABC was upregulated in M1019. Therefore, glutathione and ABC may be one factor of the insensitivity of M1019 to Cd.

## Conclusions

By comparing the differences in Cd enrichment between two varieties, we show that the insensitive varieties M1019 can concentrate more Cd in the cell, and the migration activity of Cd in the roots is relatively weak. In addition, we used proteomics analysis to study wheat-related proteins under Cd stress. Our results indicate that insensitive wheat Xinong20 can more fully utilize glutathione for detoxification, and the sensitive variety exhibits weaker DNA replication, protein synthesis and degradation. The in-depth study on the mechanism of Cd tolerance in wheat is of great significance in breeding.

## Methods

### Plant materials, growth conditions, and stress treatments

The seeds of wheat M1019 and Xi’nong20 were surface-sterilized with 1.5% (v/v) hydrogen peroxide solution for 20 min, washed with distilled water, and imbibed for 24 h. The M1019 is insensitive to Cd stress and is inbred lines crossed by Xi’nong233 and Luoxin988. The Xi’nong 20 is sensitive to Cd stress. The seeds were obtained from own laboratory. Seeds were then grown in Hoagland’s nutrient solution (pH 7) at 23 °C under 16-h light/8-h dark conditions. The culture solution was replaced every 2 days. When the second leaves of the seedlings emerged, CdCl_2_ was added to the culture solution to a final concentration of 50 μM for 24 h. Before we started cadmium stress, we selected Xi’nong 20 and M1019 plants with the uniform growth for cadmium stress. During sampling, the roots were washed with distilled water and then sampled to remove any remaining Cd from the surface Roots were individually collected and stored at − 80 °C for subsequent measurement. Three biological replications were set up in this study and the results and data analysis are based on three biological replications.

### Distribution of cd

Roots were homogenized using a mortar and pestle in a medium containing 250 mM sucrose, 50 mM Tris-HCl (pH 7.5), and 1 mM dithioerythritol. All steps were performed at 4 °C. The homogenate was centrifuged for 15 min at 3000 rpm, and the resulting precipitate included the cell wall components. The supernatant was then centrifuged for 30 min at 12,000 rpm, and the resulting precipitate included the organelle components, and the supernatant comprised the cytoplasmic component [37, 38]. After digesting each component with HNO_3_-HCIO_4_ method, and the Cd content of each sample was determined by Hitachi Z200.

### Cd chemical analysis

The extraction agents and extraction order were as follows [39]: (1) 80% ethanol: inorganic salts and amino acids, which are mainly nitrate and chloride acid salt. (2) deionized water: extraction of water-soluble organic acid salts, a generation of heavy metal phosphate. (3) 1 mol·L^− 1^ sodium chloride solution: extract pectinate, bind with protein or present a heavy metal adsorption state. (4) 2% acetic acid: the extraction of heavy metal phosphate that is insoluble in water, including second-generation phosphate and orthophosphate. (5) 0.6 mol·L^− 1^ hydrochloric acid: extract oxalate. Extraction method: 2 g of a fresh sample was placed in a beaker. Approximately 20 mL of the extraction agent was then added to keep the sample saturated, and recycled extraction after extracting it in a 30 °C incubator for 18 h. Then, an equal volume of the same extraction agent was added into the beaker of the sample, and recycled extraction for the second time after extract 2 h and repeat the extraction twice. Finally, each chemical component was extracted 4 times in 24 h, and a total of four extracts (80 mL) were pooled into a beaker. The extract of the chemical analysis of Cd was evaporated nearly 20 mL and then was determined using AAS ZEEnit700 after digesting with HNO_3_-HCIO_4_ method.

### Protein extraction, trypsin digestion, and tandem mass tags (TMT) labeling

For digestion, the protein solution was reduced with 5 mM dithiothreitol for 30 min at 56 °C and alkylated with 11 mM iodoacetamide for 15 min at room temperature in darkness. The protein sample was then diluted by adding 100 mM TEAB to urea concentration less than 2 M. Finally, trypsin was added at 1:50 trypsin-to-protein mass ratio for the first digestion overnight and 1:100 trypsin-to-protein mass ratio for a second 4 h-digestion.

For digestion, the protein solution was reduced with 5 mM dithiothreitol for 30 min at 56 °C and alkylated with 11 mM iodoacetamide for 15 min at room temperature in darkness. The protein sample was then diluted by adding 100 mM TEAB to urea concentration less than 2 M. Finally, trypsin was added at 1:50 trypsin-to-protein mass ratio for the first digestion overnight and 1:100 trypsin-to-protein mass ratio for a second 4-h digestion.

After trypsin digestion, the peptide was desalted by Strata X C18 SPE column (Phenomenex) and vacuum-dried. The peptide was reconstituted in 0.5 M TEAB and processed according to the protocol provided in the TMT kit. Briefly, one unit of TMT reagent was thawed and reconstituted in acetonitrile [40]. The peptide mixtures were then incubated for 2 h at room temperature and pooled, desalted, and dried by vacuum centrifugation.

### LC-MS/MS analysis and data analysis

The tryptic peptides were dissolved in 0.1% formic acid (solvent A), directly loaded onto a home-made reversed-phase analytical column (15-cm length, 75 μm i.d.). The gradient was comprised of an increase from 6 to 23% solvent B (0.1% formic acid in 98% acetonitrile) over 26 min, 23 to 35% in 8 min and increasing to 80% in 3 min, then holding at 80% for the last 3 min, all at a constant flow rate of 400 nL/min on an EASY-nLC 1000 UPLC system. The peptides were subjected to NSI source followed by tandem mass spectrometry (MS/MS) in a Q Exactive™ Plus (Thermo) coupled online to the UPLC. The electrospray voltage applied was 2.0 kV. The m/z scan range was 350 to 1800 for full scan, and intact peptides were detected in the Orbitrap at a resolution of 70,000. Peptides were then selected for MS/MS using NCE setting as 28, and the fragments were detected in the Orbitrap at a resolution of 17,500. A data-dependent procedure that alternated between one MS scan followed by 20 MS/MS scans with 15.0 s dynamic exclusion. Automatic gain control (AGC) was set at 5E4. Fixed first mass was set as 100 m/z.

The secondary mass spectrometry data were retrieved using Maxquant (v1.5.2.8). Parameter Settings: database search for UniProt *Triticum estivum* (136,892 series), add the library to compute random matching caused by the false-positive rate (FDR), and joined the common pollution in database library, used to eliminate the influence of protein identification results of pollution; The enzyme cutting mode was set to Trypsin/P. The number of missed cut points was set to 2. The minimum length of the peptide segment was set to seven amino acid residues. The maximum number of peptide modification was set to 5. The quality error tolerance of primary parent ions in First search and Main search was set to 20 ppm; \ and 5 ppm; \, respectively, and that of secondary fragment ions was set to 0.02 Da. Cysteine alkylation is set to fixed modification, which can be modified to oxidation of methionine and acetylation of protein N. The quantitative method was set to tmt-6plex, and the FDR for protein identification and PSM identification was set to 1%. The quantitative proteomic analysis was performed using a customer service by PTM-Biolabs Cd., Ltd. (Hangzhou, China).

## Data Availability

All data is contained within the manuscript and supplementary material. The datasets generated and analysed during the current study are available in the [ProteomeXchange] repository, [ProteomeXchange accession: PXD018179 and FTP Download: ftp://ftp.pride.ebi.ac.uk/pride/data/archive/2020/03/PXD018179].

## Abbreviations

DEPs: Differential expression proteins
MMR: Mismatch repair
TMT: Tandem mass tag
TEAB: Triethyl ammonium bicarbonate
MAPK: Mitogen-activated protein kinase
mRNA: Messenger ribonucleic acid
DNA: Deoxyribonucleic acid
LC-MS∕MS: Liquid chromatography tandem mass spectrometry
